# Exploring Age-Related Metamemory Differences using Modified Brier Scores and Hierarchical Clustering

**DOI:** 10.1515/psych-2018-0015

**Published:** 2019-06-29

**Authors:** Chelsea Parlett-Pelleriti, Grace C. Lin, Masha R. Jones, Erik Linstead, Susanne M. Jaeggi

**Affiliations:** Schmid College of Science and Technology, Chapman University, 92866 California, USA; School of Education, University of California Irvine, 92697 California, USA; School of Education, University of California Irvine, 92697 California, USA; Schmid College of Science and Technology, Chapman University, 92866 California, USA; School of Education, University of California Irvine, 92697 California, USA, Department of Cognitive Sciences, University of California Irvine, 92697 California, USA

**Keywords:** Machine Learning, Hierarchical Clustering, mBrier, Older Adults, Meta-Cognition

## Abstract

Older adults (OAs) typically experience memory failures as they age. However, with some exceptions, studies of OAs’ ability to assess their own memory functions—Metamemory (MM) — find little evidence that this function is susceptible to age-related decline. Our study examines OAs’ and young adults’ (YAs) MM performance and strategy use. Groups of YAs (N = 138) and OAs (N = 79) performed a MM task that required participants to place bets on how likely they were to remember words in a list. Our analytical approach includes hierarchical clustering, and we introduce a new measure of MM—the modified Brier—in order to adjust for differences in scale usage between participants. Our data indicate that OAs and YAs differ in the strategies they use to assess their memory and in how well their MM matches with memory performance. However, there was no evidence that the chosen strategies were associated with differences in MM match, indicating that there are multiple strategies that might be effective (i.e. lead to similar match) in this MM task.

Imagine two older adults (OAs), Grandparents A and B, with a handful of grandchildren each. Grandparent A is quite confident that he would be able to remember all of his grandchildren’s birthdays and prepare birthday presents on time. Grandparent B, on the other hand, is not as confident, and strategically marks down the birthdays on her calendar.

This scenario illustrates the concept of metamemory (MM). MM, how one thinks about one’s own memory ability, is multifaceted and various definitions exist. One dominant view breaks MM down into three components: MM *knowledge* (a person’s belief and thoughts about his/her own memory ability), memory *monitoring* (the assessment of self’s likelihood of remembering something), and memory *control* (the actions or strategies that the two previous components may lead to; see Dunlosky & Metcalfe, 2009; Dunlosky & Thiede, 2013). In our example, the Grandparents have varying beliefs (MM knowledge) regarding their ability to remember birthdays, and as they monitored and assessed their own beliefs, they arrived at two different control strategies to ensure successful outcomes (Grandparent A doing nothing and Grandparent B spending the time to write the birthdays in her calendar).

Another, not necessarily conflicting, view stems from the classic MM paper by Flavell and Wellman (1977) that treats MM as having person, task, and strategy aspects. While the person and strategy aspects map onto the knowledge and control elements of the later conceptualization of MM, the task aspects refer to the kind of materials that make it easier or harder for a person to remember. To clarify this distinction, let us return to the Grandparents. It may be easier for Grandparent A to remember the kids’ birthdays because he might not have as many grandchildren as Grandparent B does. This is analogous to having a shorter list length of elements to remember, which is an example of the *task* aspect of MM. Alternatively, at the *person* level, Grandparent A’s family could have the habit of celebrating every birthday whereas Grandparent B’s does not, thereby making birthdays more salient for Grandparent A, resulting in Grandparent A being more confident in his ability to remember the kids’ birthdays. Because of this confidence or metamemory *knowledge*, Grandparent A might not expend much energy to devise cognitive *control* or *strategies* for remembering the grandchildren’s birthdays.

## How is MM Studied?

Historically, due to the need or desire for meaningful, translational research for MM that could be applied to real life, MM has been measured via self-report questionnaires. These questionnaires may touch upon real-life scenarios that laboratory experiments cannot simulate, such as reported self-appraisal of one’s own memory in regular circumstances, the reported frequency of mnemonic strategy uses in the Multifactorial MM Questionnaire (MMQ; Troyer & Rich, 2002), memory issues and/or changes associated with healthy aging in the MM in Adulthood Questionnaire (MIA; Dixon, Hultsch, & Hertzog, 1988), or reports of how often survey respondents forget things in different situations, the seriousness and consequences of such forgetfulness, and comparison of past and present memory abilities in the Memory Functioning Questionnaire (MFQ; Gilewski, Zelinski, & Schaie, 1990). Although these questionnaires offer insights into the perceived memory abilities, or the *MM knowledge*, of participants, the lack of objective measures of MM means they do not paint a complete picture of people’s MM.

Indeed, one consistent objective among MM research is the push to go beyond merely making a judgment about the beliefs. Researchers are equally interested in the accuracies of people’s MM beliefs. This central interest may have practical value. If Grandparent A, despite the high level of confidence, were terrible at remembering the birthdays, then his poor MM would mean missed birthdays and, perhaps, disappointed grandchildren. If, on the other hand, Grandparent B were actually excellent at remembering birthdays, then her underestimation of her own memory ability would mean wasted time and, perhaps, an annoyed partner who does not understand why she is always writing things down. If we had a clearer understanding of MM in aging and, in particular, what strategies were beneficial for whom, then interventions could be tailored to meet the specific needs of individuals such as Grandparents A and B.

With the practical implications of MM, the focus of much of the most recent research on MM has rested on monitoring and control with judgment of learning (JOL) playing an important role (Dunlosky & Thiede, 2013). In JOL tasks, participants are typically asked to predict or estimate their memory performance. Though sometimes defined as “judgments of the *likelihood* of remembering recently studied items on an upcoming test” (Dunlosky & Thiede, 2013, p. 286, emphasis added), JOL tasks come in various forms. For example, in the classic MM Battery (Belmont & Borkowski, 1988), the Memory Estimation subtest that closely resembles JOL asks participants to first predict how many items they would remember from a list of 15. More recently, the field has shifted to examine JOL in a more fine-grained manner. Rather than taking JOL on the overall test level (out of all of your grandchildren, how many birthdays would you remember), researchers are increasingly more interested in JOL at the item level (e.g., how likely are you to remember grandkid 1’s birthday, grandkid 2’s birthday, and so on). For example, in a value-directed remembering task (Castel, Benjamin, Craik, & Watkins, 2002; Castel, Balota, & McCabe, 2009), participants made JOLs by placing “bets” on word items that they thought they would remember later (McGillivray & Castel, 2011). In the “bets” version, the JOL is essentially reduced to a yes/no decision.

As mentioned earlier, monitoring judgments by themselves form only one part of MM. The accuracy of these judgments is of special interest. Yet accuracy of the JOLs has also been investigated in various ways. In particular, researchers distinguish between relative accuracy (resolution) and absolute accuracy (calibration; see Schwartz & Efklides, 2012 for a discussion). Say Grandparent A has to go out shopping. For all the items they have to buy, Grandparent A is fairly confident (e.g., 80% for remembering to get eggs and coffee to 100% rating for remembering to get bread and milk) that they would remember items. Grandparent A would have low calibration if they end up remembering only half of the shopping list. However, the resolution would still be high if Grandparent A remembers the higher rated items (in this case, bread and milk) more than the lower-rated items (in this case, eggs and coffee).

Empirically, how calibration scores are calculated varies depending on the tasks and, therefore, no consistent calibration measurement exists. In the MM Battery, for instance, the accuracy of the memory estimation subtest is calculated via a somewhat arbitrary equation that weights the estimation with a separate list before the actual recall test differently from the estimation performed *after* the recall test with yet another list of items (Belmont & Borkowski, 1988). In the recent value-directed tasks (McGillivray & Castel, 2011), because the researchers’ purpose was to examine learning and strategies associated with item values and the JOL was based on a simple yes/no decision, the calibration score could only be calculated as a simple subtraction between actual number of items recalled and the number of items on which a bet was placed. To date, no MM measure has combined an objective MM task with a more fine-grained measure of participants’ own beliefs regarding their memory on any particular item, which is the aim of the present work.

### Metamemory across the Lifespan

Despite the differential trajectories of various cognitive functions across the lifespan with many memory-related functions showing age-related cognitive decline (Salthouse, 2010, Hartshorne & Germine, 2015), monitoring of MM has shown relatively little age effects. Judgments of one’s ability to remember things are notoriously difficult to measure in children and are only loosely associated with other established constructs of MM such as strategy use (DeMarie & Ferron, 2003). As people age, JOL measures have yielded much more reliable and consistent findings. When it comes to the absolute accuracy of JOLs, adults, both young and old, tend to be overconfident in their memory ability, often overestimating the number of items they can remember, though this overconfidence seems to be much more inflated in older adults (e.g., Connor, Dunlosky, Hertzog, 1997; McGillivray & Castel, 2011). Moreover, this overconfidence may be more restricted to single or initial block of trials, as there is also evidence that people can adjust their calibration based on practice, sometimes attenuating their ratings to the point of underestimating their ability in a phenomenon known as the underconfidence-with-practice (UWP) effect (Koriat, Sheffer, & Ma’ayan, 2002; Rast & Zimprich, 2009; Stine-Morrow et al., 2006; Tauber & Rhodes, 2012).

To illustrate the UWP effect, recall Grandparent A who believed that he could accurately recall the birthdays of all his grandchildren without any external support such as a calendar or reminders. At a family gathering, his son Erik decided to test out this claim by asking Grandparent A to list all the grandchildren’s birthdays. Contrary to what Grandparent A believed, he was able to remember the majority of the birthdays but not all of them. Although he was fairly well calibrated, Grandparent A may announce at the next family gathering that he could probably only remember half of the grandchildren’s birthdays. This time, however, he was actually able to remember more birthdays than the previous time. He was overconfident the first time, but became underconfident afterward. He has experienced the UWP effect. Though this scenario is simplistic for illustrative purposes (e.g., only two “trials” and only one rating for the entire list rather than individual rating for each grandchild), numerous studies have documented the UWP in multiple, arguably more complex conditions (e.g., Koffer et al., 2002; Stine-Morrow et al., 2006; Tauber & Rhodes, 2012). For example, conducting a grand analysis across 11 studies and extending these studies further, Koriat and colleagues (2002) established the robustness of the effect: UWP was found whether feedback was given or not, across varying incentive levels, study time, and list types (e.g., word-association list or simple word list), just to name a few.

Nevertheless, exceptions do exist for the robust UWP effect (e.g., McGillivray & Castel, 2011; Rast & Zimprich, 2009). For example, in the novel paradigm where participants made judgments to “bet” on the likelihood of remembering words based on their assigned values, neither older nor younger adults became underconfident in later word lists (McGillivray & Castel, 2011). It should be noted that participants did indeed lower their number of bets in subsequent lists and became more calibrated later on, but they never remembered more words than they bet on (McGillivray & Castel, 2011). This surprising lack of UWP could possibly be related to the novel “betting” paradigm where the binary yes/no decision and its accuracy could mean more or fewer points in the final score. More research using this “betting” paradigm would therefore be beneficial in addressing some of these discrepancies.

In addition to the initial overconfidence (albeit to different degrees) as measured by absolute accuracy of their monitoring judgments, YAs and OAs display similar patterns in monitoring relative accuracy (e.g., Hertzog et al., 2002; 2010; Robinson et al. 2006; Tauber & Rhodes, 2012; Stine-Morrow et al., 2006). For example, in experiments with word-pair associative learning tasks with an explicit instruction to form and use mental imagery for the word pairs, YAs and OAs based their JOLs on whether they were able to successfully form an image (there was no age difference in imagery formation success), suggesting that both YAs and OAs were effective in monitoring their memory and strategy—image formation—use (Robinson et al., 2006). Similarly, gamma correlation measures between JOLs and recall showed both YAs and OAs were equally accurate in monitoring their memory of texts that they read (Stine-Morrow et al., 2006).

Nonetheless, some earlier studies showed that OAs use monitoring to a lesser extent than YAs do (Dunlosky & Connor, 1997; Souchay & Isingrini, 2004). Additionally, even though the UWP effect has been shown in both age groups, sometimes OAs do not display the underestimation following learning in the first trial (Rast & Zimprich, 2009). In two experiments varying in the number of trials (two trials only in experiment 1 and five trials in experiment 2), both YAs and OAs overestimated their ability to remember word pairs during the first trial. However, only YAs underestimated in the subsequent trials despite improvements in estimation in both groups (Rast & Zimprich, 2009).

Beyond memory monitoring, it appears that YAs and OAs also share methods of memory control or strategy (Tauber & Witherby, 2015), though some patterns of differences have also emerged. One classic method of investigating individuals’ cognitive control or strategy use is to have participants make decisions regarding how they would allocate study time (e.g., Castel et al., 2002; Dunlosky & Hertzog, 1997; Metcalfe, 2002; Price et al., 2010; Price & Murray, 2012). Across different studies that varied the items in terms of difficulty or values (i.e. points awarded), two patterns emerged. First, both YAs and OAs tended to prioritize easier items over harder items. Second, both groups tended to prioritize high value items (e.g., Castel et al., 2002; Price et al., 2010). However, OAs were only likely to prioritize high value items that were also easy, whereas YAs were more likely to prioritize high value items regardless of difficulty. This strategy difference may be related to OAs’ lower memory self-efficacy (Price et al., 2010). Furthermore, studies demonstrated that in learning a novel calculation task, OAs were less likely and slower to switch from computing to retrieval strategy after repeated exposures to the same stimuli (Touron, Hoyer, & Cerella, 2004). Similarly, OAs were less likely to use retrieval as a strategy in noun-pair associative learning tasks (Rogers, Hertzog, & Fisk, 2000).

Thus, there seem to be subtle differences between YAs and OAs in various aspects of MM. Still, while the literature on MM in OAs has been developing for some time now, there is no consensus regarding whether MM accuracy is impacted by aging, or whether specific strategy use might play a role in any differences or the lack thereof. Furthermore, the literature appears fairly settled on the analytical approaches to MM, employing straightforward deviation scores (e.g. Brier scores) for calibration, and gamma correlations for resolution. Though the distinction of absolute versus relative accuracy is imperative as they answer different questions pertaining to different underlying metacognitive mechanisms (calibration pointing to judgment precision and resolution to the correspondence between judgment and performance; see discussion in Schraw, 2009), the two measures may sometimes be at odds with each other, making an overall inference about one’s MM difficult. For example, the robust UWP effect exists *only* for absolute accuracy (calibration); in the studies that demonstrated UWP in calibration, participants’ resolution actually improves in later blocks or presentations of trials (e.g., Koriat, Ma’ayan, Sheffer, & Bjork, 2006). Considering the discrepant findings for calibration and resolution, a hybrid score may be useful in enhancing our understanding of MM and any age-related differences.

In order to conceptualize a “new” approach to examine MM data, we will provide a brief overview of the traditional, established methods in the following.

### Calibration

Calibration, or absolute accuracy of the participants’ judgment as compared with their actual performance, is typically a deviation score calculated via subtraction between performance and judgment. Sometimes this subtraction is done in a straightforward manner (e.g., Koriat et al., 2006; McGillivray & Castel, 2011), while other times researchers calculate calibration using equations that assign different weights to different lists (e.g., Belmont & Borkowski, 1998). Among the varied methods of calculating calibration, one measure (and its variants) stands out and is most commonly used: Brier score (in MM literature, also known as calibration index; see Schraw, 2009):

$$\frac{1}{n}\sum_{i=1}^{n}{\left(acc_{i}-jol_{i}\right)}^{2}$$

Brier score measures the accuracy of probabilistic predictions (Rufibach, 2010) and provides the precision of the confidence ratings (i.e. JOL). As the equation would suggest, a score of zero corresponds to perfect accuracy (imagine JOL of 100% and performance of 100%, (100–100)^2 = 0) and a score of one would be no accuracy (for example, a 100% JOL and 0% performance). Thus, counterintuitively, a higher score is considered having “worse” MM using this index. The precise nature of this score also comes with another caveat: individuals may have internal differences in providing confidence ratings. For example, cross-cultural studies of responses on Likert scale surveys revealed that Asian and Asian American participants are less likely than other ethnic groups to mark the extreme values (e.g., Batchelor & Miao, 2016; Chen, Lee, & Stevenson, 1995). Thus, two people who are equally confident may place their ratings based on different internal scales despite being given the same scale of, say, 0–10, and Brier score does not correct for potential scaling differences.

### Resolution

This caveat of absolute scores can be addressed by examining participants’ relative accuracy, or resolution. In MM research, gamma correlation (Nelson, 1984) is most commonly used to examine how well participants’ judgments correspond with their actual performances (e.g., Koriat, 1997; Koriat & Goldsmith, 1996). Because the correlation is largely contingent upon variability among the ratings and performances, cases with extreme scores (e.g., JOLs of all 100% or 0 or 100% accuracy) had to be excluded. While this does not interfere with the theoretical validity of gamma, it can present practical issues. In the current dataset, around 17% of gamma values were non-computable. Because of this artifact and because participants’ performance tends to become better throughout an experiment, resolution scores from one block to the next are often calculated based on dwindling sample sizes (see for example Koriat et al., 2006).

### Discrimination

Yet another dimension in MM studies is the concept of discrimination, or the extent to which confidence ratings between correct and incorrect items differ and can be distinguished from one another (Schraw, 2009). Positive discrimination scores would indicate that participants were more confident on items they recalled correctly than non-recalled items. Conceptually, discrimination would be an ideal, additional construct to measure metacognitive awareness. However, as the comparison would be between correct and incorrect items (rather than within item JOL and accuracy comparison as in the case of calibration), discrimination scores are calculated at the aggregate level and may be less precise.

Resolution and discrimination scores have been instrumental for theory development (e.g., cue-utilization theory), providing insights into the mechanisms with which people make confidence ratings or monitor their own knowledge or memory. Yet, the addition of a hybrid score may address some practical concerns, ranging from something as trivial as answering participants’ questions of “I feel like I did worse later. Am I right?” to something more substantial as addressing the cases when the data do not allow for proper, meaningful calculation of resolution scores. Having a hybrid score that takes into account both the precision *and* association between judgment and performance may be helpful as a first-step presentation of a birds-eye view of the metamemory scheme before breaking down into the details of the mechanisms with which people monitor their knowledge and memory.

To address these issues, our study employs a novel version of a MM task that allows for a more detailed assessment of participants’ own beliefs regarding their memory on any particular item. Further, we use machine learning methods to understand nuances in the data that may shed light on these issues in a way that traditional analytical methods have not been able to in the past. To do so, we take advantage of the fine-grained nature of the individual word bets. Rather than having participants estimate their memory at the list level, providing judgment ratings at the item level allows for a more nuanced understanding of MM. We seek to answer the question of whether older and younger adults differ in their MM, as measured by a new hybrid, mBrier score, and how their strategy use might affect the new hybrid MM mBrier scores.

## Method

### Participants

Data was collected from 233 YAs and OAs. Healthy OAs were recruited through flyers distributed in community centers in southern California, and they received monetary compensation for their participation. YAs were undergraduate social science students who participated for course credit. Data for all participants were collected in a controlled laboratory setting. Sixteen (n = 13 OAs; n = 3 YAs) participants were excluded due to technical difficulties, or missing/corrupted data. Listwise deletion was used for missing data due to the restrictions imposed by our clustering methods. The final analytical sample consisted of 79 OAs (mean age = 73.72, SD = 4.91; 62 women; vocabulary score 22.08, SD = 3.89) and 138 YAs (mean age = 20.71, SD = 2.38; 101 women; vocabulary score 15.44, SD = 3.65).

### MM Task

This MM task was adapted from a similar computerized task by McGillivray and Castel (2011). Participants were presented with five rounds of 12 words, shown one at a time with the overall instruction to remember as many words as possible. After each word was shown for 3 seconds, participants were given up to 5 seconds to place a bet (a version of a JOL) between 0 and 10 points. After seeing the 12 words, participants were asked to recall as many words as possible by typing them into the computer. They were told that if they correctly remembered a word, the bet for their word would be added to their score. If they did not remember a word, their bet would be subtracted from their score. Participants were given unlimited time to recall the words of each list. Extra words that were entered (i.e. words not in the list) were not counted in their score. Correct spelling and tense were required in order to be counted as correct, however participants were allowed to correct misspellings if they noticed them. After each round, participants were presented with their score—the MM score—for that round before proceeding to the next round of 12 words. The experimenter further explained that the objective is for the participants to get as high of a score as possible.

### Word Selection

One version of the word list was adapted from McGillivray and Castel (2011). For the other, we combined the sets of words from the English Lexicon Project (Balota et al., 2007), which contains the Hyperspace Analogue to Language (HAL) word frequency norms (Lund & Burgess, 1996) from the HAL corpus of about 131 million words, with databases containing valence (Warriner, Kuperman, & Brysbaert, 2013) and imageability (Bird, Franklin, & Howard, 2001). Only words with ratings for these lexical features remained in the potential stimuli pool. We further limited the stimuli to 4–7 letter words that are nouns, neutral in terms of emotional valence (1 standard deviation around the median of valence), high frequency (1 standard deviation around the 75^th^ percentile of the frequency index), and neutral imageability (1 standard deviation around the median). To create the second version, we randomly selected 60 of the words and split them into 5 lists . As mentioned earlier, each word list contains 12 words (therefore each version has 60 words). Within sets, every participant received the same five lists in the same order. However, participants were randomly assigned to receive either set A or B.

While there is a significant difference between the number of correctly recalled words between the two versions (p = 0.02, Bayesian analysis did not provide strong support for a difference, with a BF_10_ = 0.986) as well as differences in frequency, valence, concreteness, imageability and length (p’s < 0.01, BF_10_’s > 13, all BF_10_’s but valance > 192), there was not a significant difference between the average bets nor mBrier scores (score described below), arousal, nor polysemy (BF_10_’s < 0.827). Exact summary statistics are available in the Supplementary materials. Within each version, there is no significant effect of round (1–5) or interaction between version and round in any word characteristics, signifying that within versions, the word lists for round do not differ significantly. Furthermore, for all clusters examined in this paper, there was no statistically significant difference between the distributions of version between clusters (i.e. clusters did not have significantly different proportions of either version) Bayesian analysis agreed, finding no strong evidence that there is a difference in distribution between the two versions (all BF_10_’s < 0.86 ).

### mBrier Score

The MM score as described above, is a measure of both MM and raw memory capacity, and, along with the number of words recalled irrespective of bets, has been used as the primary dependent variable for that measure (McGillivray & Castel, 2011). Participants with high scores must both have good MM *and* be able to remember some words, since the only way to gain points is to correctly recall a word. While this specific combined measure is useful, there is also a need to tease apart the memory capacity and MM components of this score. This paper offers a different, supplementary score, called the modified Brier score (mBrier) that offers better insight into the MM component of the task. We will provide vignettes and general descriptions of when mBrier offers better or more practical scores over two traditional measures of metamemory performance: Gamma and a traditional Brier score.

The mBrier score is a hybrid score (for a description of hybrid scores, see Schraw, 2009). Its calculation follows the traditional Brier score calculation. However, instead of using binary JOLs, or even continuous percentages (e.g. the numerical response to “what is the probability that you will remember this word?”), the mBrier score uses a scaled and ranked transformation of the JOLs. Bets/JOLs ranked from 1 to m, with m being the number of non-zero bets/JOLs.

$$\frac{1}{n}\sum_{i=1}^{n}{\left(acc_{i}-Rjol_{i}\right)}^{2}$$

*where Rjol*_*i*_ *is the ranked jol,where rank is calculated after excluding all jols*=0

In order to calculate the ranked JOLs, first, all items that were given a JOL of 0 are excluded, and left as 0’s. Then, the remaining items’ JOLs are ranked. The resulting ranked JOLs are then scaled by the maximum rank in order to get a probability between 0 and 1.

Traditionally, many MM studies have used the Goodman-Kruskal gamma as a measure of resolution.

The formula for gamma is shown below for ease of reference.

$$G=\frac{N_{s}-N_{d}}{N_{s}+N_{d}}$$

Where *N*_*s*_ is the number of concordant word pairs (e.g. where the bet of word A is higher than the bet of word B, and the accuracy of word A is higher than word B) and *N*_*d*_ is the number of discordant pairs (e.g. where the JOL of word A is higher than the JOL of word B, and the accuracy of word A is lower than word B). In this calculation, all pairs where either the JOL or accuracy are the same (e.g. if a subject recalled or did not recall both words, or gave the same JOL for both words) are discarded.

While Gamma is generally a useful resolution measure, it can be lower when JOLs are not binary (Koriat, 1997). There have also been concerns about reliability of Gamma (e.g., Kelemen, Frost, & Weaver, 2000; Thompson & Mason, 1996) and how Gamma appeared unrelated to task difficulty and individual differences (Maki, Shields, Wheeler, & Zacchilli, 2005). The lack of reliability could be related to how many item pairs are excluded in the Gamma calculation. This is of particular concern, as patterns are not noncomputable at random, rather, certain patterns are more likely to be excluded, such as bet/JOL perseveration.

This is increasingly impactful towards the extremes of the Gamma score (−1 and 1). The modified Brier score is highly negatively correlated with Gamma (r = −0.71 in this sample; correlation is negative because Gamma and mBrier are coded differently with high Gamma scores and low mBrier scores both indicating good performance), however it shows the most difference at the extremes. The negative correlation is due to the fact that Gammas score from −1 to 1 with 1 being the highest performance, while Brier scores go from 0 to 1 with 0 being the highest performance. An example from our dataset of where the modified Brier score allows better differentiability between MM performance is presented below.

Participants A and B (data is pulled from our dataset) both have a Gamma of 1 (the highest score possible), however they score very differently using a modified Brier score (0.585 and .183 respectively).

There is a clear difference in the performance of these two participants, yet this difference is not captured by Gamma. Participant A gives maximum JOLs for all but 1 word, and only recalls 4 of them. However, because 11 out of the 12 JOLs are the same (10), the number of pairs that Gamma considers for Participant A is severely limited. Since there is only one low JOL, we can only consider pairings that include the word associated with this JOL. Since it was not recalled, we also must exclude pairings with words that were not recalled. In this case, it leads to a situation in which the one non-recalled word with a low JOL (“owl”), is only compared to recalled words. This leads to exclusively concordant pairs (owl-girl, owl-frog, owl-bus, owl-apple), and thus a high gamma. However, examining Participant A’s strategy reveals that for the most part, they are not good at appropriately assigning JOLs, they happened to have one case in which they did appropriately assign a lower JOL to a non-recalled word.

On the other hand, Participant B also has a Gamma of 1, however it is clear from the strategy of Participant B, that they have a better grasp of giving appropriate JOLs. While they did recall one low JOL word (“help”), overall their JOLs are high for recalled words and low for non-recalled words. Their betting pattern allows mBrier to reflect this, unlike with Participant A.

Similarly, two participants, C and D (again, pulled from our dataset), both have a Gamma of −1. However, their scores using our score are quite different (0.679 and 0.570 respectively).

In the case of Participant C, the Gamma score and our score coincide: both give it an a poor score (−1 is the lowest possible Gamma), indicating that JOLs are inversely correlated with accuracy. If you give a low JOL, you’re most likely to recall the word. Participant D got the same Gamma as Participant C, however, looking at their performance, it is possible to see that while they were more likely to remember words with low JOLs, the magnitude of the inappropriateness of their JOLs is smaller, reflected in our score of 0.57.

Differentiating between these two patterns is important, and the authors believe that a single score reflecting potential discrepancies is useful, even though it is valuable to look at *calibration* and *resolution* separately and does not replace Gamma. Our hybrid score represents a comprehensive overview of performance that allows for finer grained differentiation. This is especially valuable at the extreme ends of Gamma Scores.

Importantly, and specifically to our task and dataset, Gamma has an undefined value when either all JOLs are the same, or all accuracies are the same (either all words recalled or none recalled). Unfortunately, this scenario happens often, specifically, in about 17% of rounds in our sample. Since full feature vectors are needed to perform good hierarchical clustering, using Gamma’s reduces the data set from 217 to 139, signaling that at least 78 of the original 217 participants had at least one noncomputable gamma value.

### Brier Scores

Brier scores are the mean squared error of JOLs (taken as a probability) compared to accuracy of recall or recognition. Brier scores also are computable on an item-by-item level. A comparison with mBrier which is also computable on an item-by-item level is discussed below. The formula for Brier score shown below are sometimes referred to as the absolute accuracy (calibration) index (see Schraw, 2009).

$$\frac{1}{n}\sum_{i=1}^{n}{\left(f_{i}-o_{i}\right)}^{2}$$

where *f*_*i*_ is the preditcted probability of success between 0 and 1. And o_i is the accuracy,0 or 1.

As a measure of calibration, Brier scores treat JOLs as probabilities of recall. However, different participants often use the scale in this task differently. This is especially important in this task, where JOLs were framed as “bets.” In order to account for this, the modified Brier score uses the scaled rank of the JOL rather than the unranked JOL in its calculation. The following vignette is an example of when mBrier is helpful, and was chosen specifically to elucidate more clearly when mBrier is beneficial. Two participants, Participants E and F (data extracted from our study), could have a similar MM performance, but because of their different uses of the JOL scale, have different Brier Scores.

By using a modified, ranked Brier score, information above and beyond calibration alone can be observed. In the above case, both participants would receive a modified Brier score of 0.18. In this respect, our modified score shares characteristics with both calibration and resolution. Again, this is especially important in the current task because JOLs were framed as bets rather than percentages. Derived from the classic absolute accuracy index (i.e. Brier score), our modified score utilizes rank order, resulting in scores that would highlight relative accuracy. Our score aims to account for differences in scale usage by using the ranked value of bets.

## Analytical Approach

For strategy clusters, the raw bets for all 60 (5×12) words were used, resulting in a 60-dimensional vector. For mBrier clusters, the mBrier score for each round was used, resulting in a 5-dimensional vector.

### Hierarchical Clustering

Hierarchical Agglomerative clustering was used to cluster both the raw betting and mBrier data at the participant level. Hierarchical Clustering reveals the hierarchical structure of groups within the data, allowing for more both coarse and fine-grained analyses of different strategies and calibration improvements. In Hierarchical Agglomerative clustering—also called bottom-up clustering—each data point starts off as its own singleton cluster. Each cluster is then continually merged with the next closest cluster until all the data points are in one cluster (Ward, 1963). Multiple linkage criteria exist to determine the distance between two clusters. In this analysis, complete-linkage was used. The results of Hierarchical Agglomerative clustering can be shown visually using a dendrogram that shows the nested structure of the clusters. Hierarchical clustering allows for the examination of multiple levels of nested clusters.

Hierarchical clustering was performed on 60 dimensional vectors of participant’s bets across all 60 trials. For mBrier scores, 5 dimensional vectors of average mBrier scores by round were used. Using unsupervised machine learning methods like hierarchical clustering can help detect naturally occurring groups in the data. For example, participants who use different betting strategies, or participants who have similar trajectories for improvement of their mBrier scores. We believe that looking at the hierarchical structure of clusters will be beneficial because it will allow for the examination of similarities between different clusters. This will allow researchers to look at as coarse or fine-grained clusters as necessary. In some situations, it may be beneficial to look at a high level, coarser clusters, especially in the case that clusters are used as a basis for targeted interventions. However, looking at lower level, more fine-grained clusters may be useful when looking at individual differences. The highest level clusters (2 and 3 clusters) were examined in the present paper in order to pull out high level differences between different strategies and performances.

### Application to our Dataset

Data for all participants, regardless of age were clustered on both their bets and their mBrier scores over time. This helps distinguish groups of participants who have either similar strategies when completing the task, or similar improvements over time, i.e. across the 5 rounds. The betting strategy groups are of special interest since it will allow us to a) define and describe different strategies used on this task, and then b) investigate whether OAs use different strategies than YAs. We can also test whether there is a differential effect of strategy use on mBrier score (i.e. whether certain strategies are associated with higher mBrier). Understanding the different strategies and their association with lower mBrier may pave the way for interventions that teach strategies that are helpful for OAs.

## Results

Summary Statistics for the data are shown in Table 2. Data were analyzed using R (3.4.3 Kite-Eating Tree) and JASP (JASP Team (2018). Version 0.8.3.1).

### Bets and Correctly Recalled Words by Age

Average bets across all 60 words was not significantly different between OAs and YAs (*t*(215) = 1.769, *p* = 0.078, d = 0.25; BF_10_ = 0.661). However, as expected, the average number of correctly recalled words was significantly different between OAs and YAs, with OAs remembering less words (*t*(215) = 11.06, *p* <0.001, *d* = 1.56 ; BF_10_ = 3.651e19).

### mBrier Score by Age

**Figure 2.**
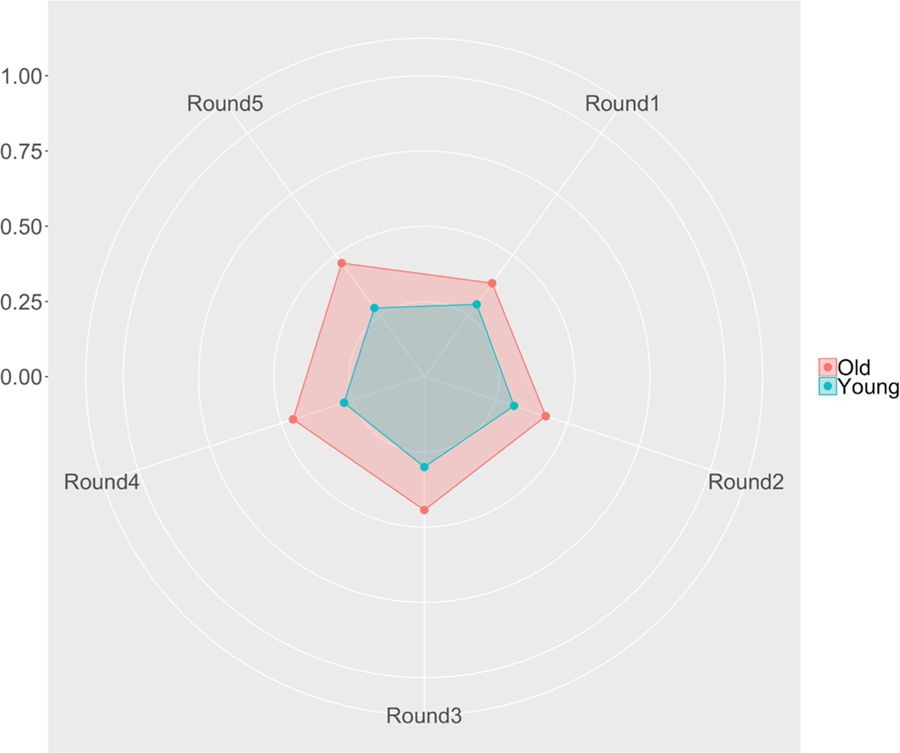
A radar graph showing the average mBrier score for OAs (red) and YAs (blue) across all 5 rounds of the MM task. Lower scores are better. The scale on the left shows the progression of values from the center of the circle, outwards.

OAs had a significantly worse average mBrier score than YAs (*t*(215)= −7.681, *p* < 0.001, *d = −1.084*, BF_10_ = 1.152e10). The hybrid mBrier scores across rounds—instead of overall averages—can be seen plotted in Figure 2. In order to test whether age differences emerge over time, we ran a repeated measures ANOVA with age group as between subject factor and round as within factor, which revealed a significant age group × round interaction, agreeing the with Bayes Factor for this analysis (*F*(4,860) = 4.53, *p* = 0.001; BF_interaction_ = 9.77).

### Hierarchical Strategy Clustering

**Figure 3.**
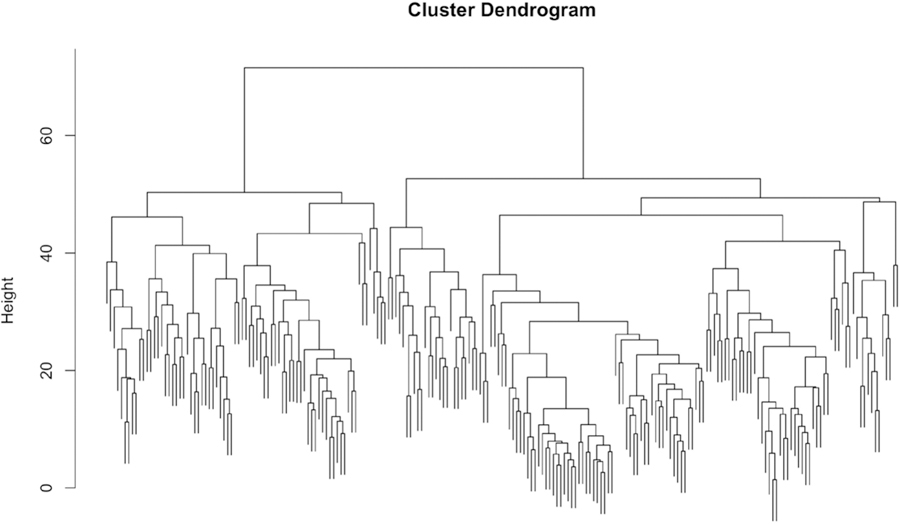
Dendrogram from hierarchical clustering of betting strategy data at the participant level showing the relative distan-ces between clusters (Height) and their hierarchical structure. The distance between clusters visually represents how different clusters are from one another, whereas the hierarchical arrangement shows how clusters are nested within one another.

Figure 3 shows the dendrogram for the hierarchical clustering using the strategy data. Dendrograms show the relative distance between clusters, as well as indicate the hierarchical arrangement of the clusters. The distance between clusters visually represents how different clusters are from one another, whereas the hierarchical arrangement shows how clusters are nested within one another.

This dendrogram reveals less separable clusters (compared to dendrograms with more density at the bottom) since the dendrogram is denser in the middle range of the y-axis than at the bottom. Typically, clusters that are cohesive and separate (i.e. items in a cluster are very close to other items in that cluster, and far away from items in other clusters) will have dendrograms that are denser at the lower range of the y-axis (i.e. more connections will be made in the lower range of the y-axis).

### Two Strategy Clusters

First, we look at the two highest level clusters: Cluster 1 consisted of relatively low bets and low variance between bets. Instead, Cluster 2 consisted of high bets, and higher variance. These patterns can be seen more clearly in the Radar graph in Figure 3a.

**Figure 4.**
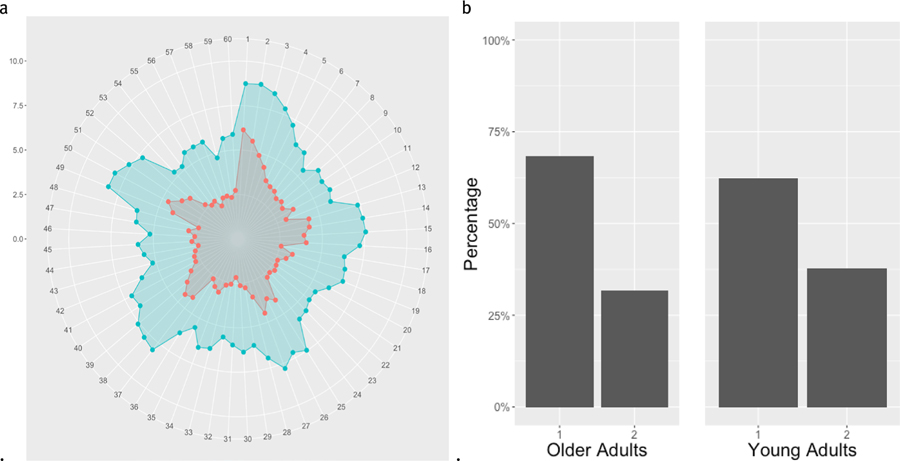
a. Radar Graph displaying bets for the two-cluster analysis. The first cluster (low variation and low average bets) is shown in red, and the second cluster (higher variation and higher average bets) is shown in blue. The Radar graphs display the mean bets with each spoke representing the bets for an item. Items are read clockwise from the 12 o’clock position onward. The scale on the left shows the progression of values from the center of the circle, outwards. b. Barplot showing counts of OAs (left) and YAs (right) for the two strategy clusters shown in a. Cluster 1 represents low bets/low variance, Cluster 2 represents high bets/high variance.

Figure 4b displays the distributions of OAs (left) and YAs (right) between these two clusters. A chi-square test of independence revealed no significant difference in strategy usage between OAs and YAs. (χ^2^ (1)= 0.5575,*p* =0.4553; *BF*_10_ 0.249; *Fisher’s Exact Test, p*=0.3815 ). Fisher’s Exact Test was run for all analyses since some cluster assignments resulted in an expected cell value smaller than 5.

### Three Strategy Clusters

**Figure 5.**
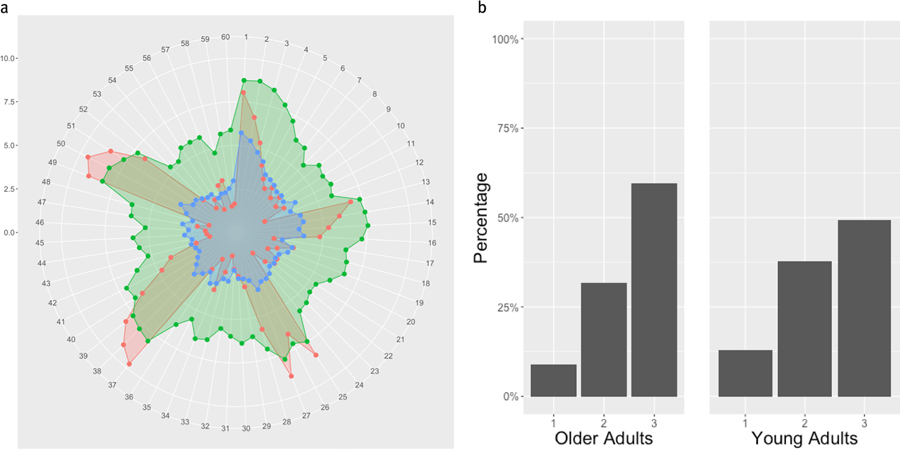
a. Radar Graph displaying bets for the three-cluster analysis. The third cluster (low bets/ low variance) is shown in blue, and the second cluster (high bets/high variance) is shown in green, and the first cluster (high variance and more cyclical betting patterns) is shown in red. The scale on the left shows the progression of values from the center of the circle, outwards. b. Barplot showing distribution of OAs (left) and YAs (right) between the three strategy clusters shown in a.

Clustering the betting strategy data into three clusters again yielded the same cluster with relatively low bets and low variance between bets, which we will now refer to as Cluster 3 (blue). And Cluster 2—with high bets, and higher variance—remained largely the same (green). Cluster 1, is characterized by extreme variance, and a cyclical pattern between each of the 5 rounds (note the 5 spikes in red in Figure 5a). Clusters 1 and 3 in this analysis were previously clustered together in the 2-cluster analysis above. These patterns can be seen more clearly in the Radar graph in Figure 5a.

The distributions of Older and YAs between these clusters is shown in Figure 5b. Both a chi-square test of independence and a Fisher’s exact test again did not reveal a significant difference in strategy usage between OAs and YAs (*χ*^2^ (2)= 2.268, *p* =0.3217; *BF*_10_ =0.105; *Fisher’s Exact Test,p* =0.3276).

### mBrier Clusters

The present paper will focus on analysis with 2 and 3 clusters, however, analyses using 4–9 clusters can be made available by emailing the authors.

The dendrogram for the hierarchical clustering of mBrier scores is dense at the bottom, revealing more separable clusters since points tend to join together in the lower range of the y-axis. On the lefthand side of the graph, a smaller cluster that is far away from the rest of the data can be seen (as it connects to the rest of the data very high on the y-axis).

### Two mBrier Clusters

**Figure 7.**
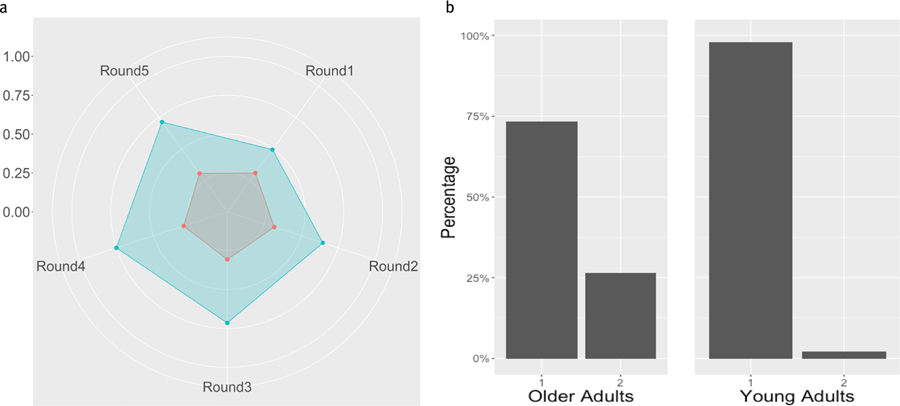
a. Radar Graph displaying mBrier scores for the two-cluster analysis across the five rounds. Cluster 1 (stable, low mBrier) is shown in red, Cluster 2 (higher, less stable mBrier scores) is shown in teal. There are five spokes in the mBrier radar graph with each spoke representing the average mBrier scores for each round. mBrier can be between 0 (the center of the graph) and 1 (the edge of the graph) with lower scores being better. The scale on the left shows the progression of values from the center of the circle, outwards. b. Barplot showing distribution of OAs (left) and YAs (right) between the two mBrier score clusters shown in a).

In the 2-Cluster analysis, the first cluster, Cluster 1 has a relatively stable mBrier score over all 5 rounds, and mBrier scores were relatively low (indicating higher performance) compared to the other cluster. Cluster 2’s mBrier scores were slightly more variable and characterized by many ups and downs, and on average were higher (worse) than the mBrier scores of Cluster 1. These patterns can be seen in the radar graph in Figure 7a.

The distribution of OAs and YAs between these two clusters was significantly different (*χ*^2^ (1)= 28.00, *p* = <0.001; *BF*_10_= 314124; *Fisher’s Exact Test, p* <0.001) , suggesting that OAs and YAs tend to differentially adjust their mBrier scores across the five rounds. Compared to YAs, OAs are more likely to be in Cluster 2, which has higher, less stable mBrier scores (cf. Figure 7b). This pattern is also supported by the difference in mBrier as a function of age seen in Figure 2.

### Three mBrier Clusters

**Figure 8.**
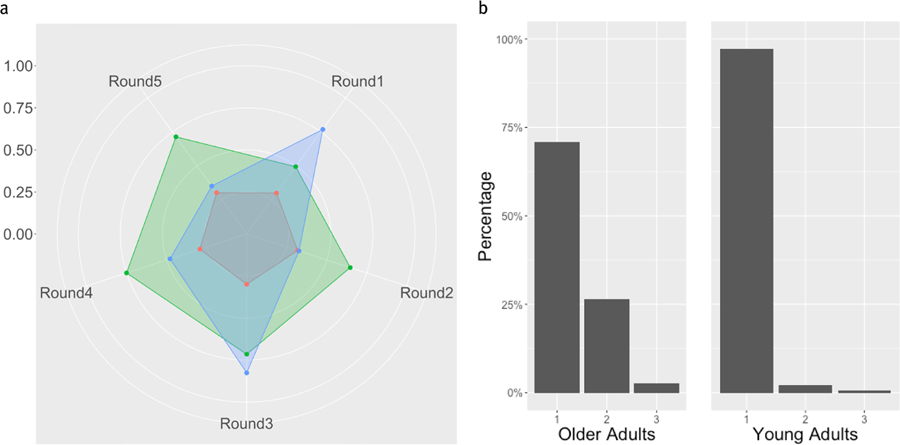
a. Radar Graph displaying mBrier scores for the three-cluster analysis. Cluster 2 remained the same from the two-cluster analysis. Cluster 1 (with stable, low mBrier scores across rounds) and 3 (blue) were previously clustered together in the 2-cluster analysis (Cluster 1 shown in red, Figure 7a). Cluster 3 has more unstable mBrier scores across rounds. The minimum mBrier score (0) is displayed at the center, with increasing mBrier scores moving outward. The scale on the left shows the progression of values from the center of the circle, outwards. b. Barplot showing distribution of OAs (left) and YAs (right) between the three mBrier clusters.

In the 3-Cluster analysis, Cluster 2 from the 2-Cluster analysis remains the same. Cluster 1 from the 2-cluster analysis breaks into Clusters 1 and 3. The clusters are visualized in Figure 8a. Cluster 1, the red regular pentagonal cluster, again contains those with relatively low, stable mBrier scores across all five rounds. Both Cluster 2 (green) and 3 (blue) show higher (worse performance) overall mBrier scores, as well as more variation between rounds.

The distribution of OAs and YAs between these 3 clusters, shown in Figure 8b, was significantly different *(χ*^2^ *(2) = 32.19, p <0.001; BF*_10_ *= 64135; Fisher’s exact test, p <0.001)*, again suggesting that OAs and YAs tend to differentially adjust their mBrier scores over time.

### mBrier Score by Strategy Cluster

**Figure 9.**
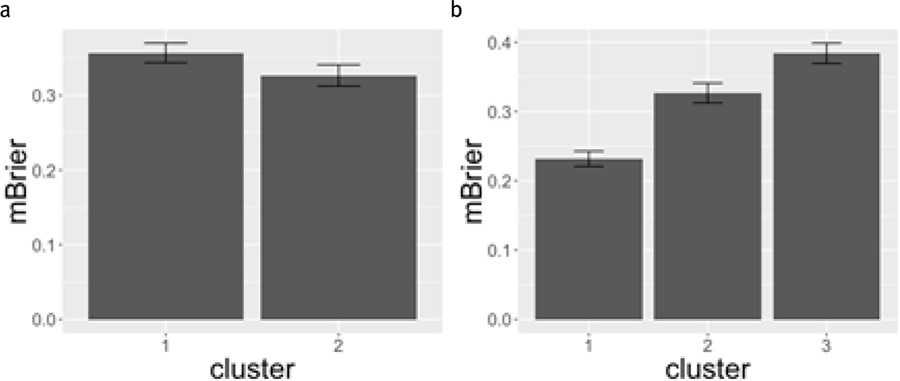
Mean plots of mBrier score by two (a) and three (b) cluster strategy. Error bars represent +/− standard errors of the mean.

For the two strategy cluster analysis, there was no significant difference between the two clusters’ average mBrier score (*t*(215) = 1.459, *p* = 0.146, *d* = 0.207, BF_10_ = 0.417). This result indicates that there is little evidence that strategy use between the two clusters has an effect on overall mBrier scores. Figure 9 displays the mean mBrier scores for the two-strategy clusters (left panel) and the three-strategy clusters (right panel). However, there was a significant difference between the average mBrier score of the three strategy clusters (*F*(2,214) = 13.61, *p* < 0.001 , = 0.113, BF_10_ = 5054.46), indicating that between these three strategy clusters, there is a significant difference between the average mBrier score, with Cluster 3 (low bet, low variation) having the worst overall mBrier scores, and Cluster 1 (cyclical pattern bet) having the lowest (best performing) mBrier scores.

**Figure 10.**
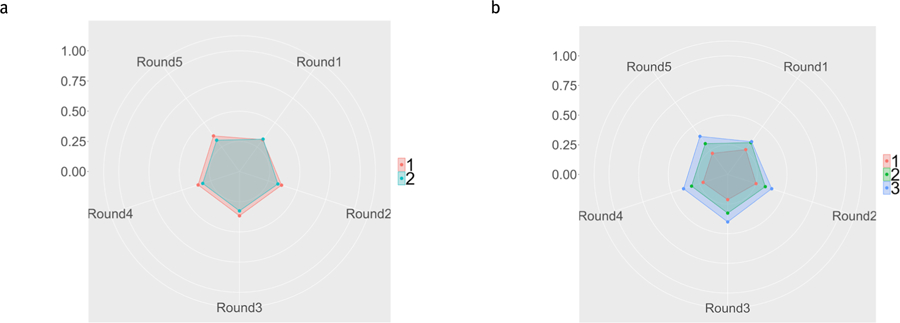
Radar Graphs displaying the average mBrier score between the two (a) and three (b) strategy cluster groups as a function of round. Lower mBrier scores are closer to the center of the graph and represent better performance.

In order to visualize the differences in mBrier scores between the clusters, radar graphs were created. The left panel of Figure 10 shows the radar graph for the mBrier scores with the two strategy clusters, and the right panel shows the graph with three strategy clusters.

Overall, the average mBrier scores for each of the five rounds is relatively similar for the two cluster groups. However, in the three cluster groups, a clear pattern of lower (better) mBrier scores for Cluster 1 emerges.

### Bets and Accurately Recalled Words by Strategy Cluster

Both average bet and average number of correctly recalled words were significantly different between the two Strategy clusters (*Bets*: *F*(1,215) = 374.1, *p* < 0.001; *BF10* = 1.919, *Accurately Recalled Words*: F(1,215) = 22.05, p < 0.001; *BF*_10_ = 3217.94), indicating that certain strategies maybe be used differentially by people, which might be related to individual differences, such as memory capacity. The same pattern was found in the three cluster analysis (*Bets*: F(2,214) = 201.6, *p* < 0.001; *BF*_10_ = 3.96 e46, *Accurate Recalls*: *F*(2,214) = 12.21, *p* < 0.001; *BF*_10_ = 2761.21). Post-hoc Tukey’s HSD tests revealed a significant difference in average number of correctly recalled words between Clusters 2 and 3, indicating that Cluster 2 had a significantly higher number of accurately recalled words (p <0.001), and a significant difference in bets between all three clusters with Cluster 3 having significantly lower average bets (p < 0.003).

## Discussion

Age-related decline in cognitive functioning has been well-documented. Indeed, in our study, OAs recalled significantly fewer words overall, indicating poorer memory capacity. However, it is less clear whether MM also declines with age (Bruce et al., 1982; Salthouse, 2010). While we replicated the well-documented finding that OAs demonstrated poorer memory ability as compared to YAs, the average bets across all 60 words was not significantly different between OAs and YAs. Since the bets/JOLs of the OAs were on average as high as those of the YAs who demonstrated better memory performance, and mBrier scores were significantly lower for YAs, we can conclude that the OAs exhibited poorer MM, and displayed the overconfidence phenomenon that has been described before (e.g., Connor, Dunlosky, Hertzog, 1997; McGillivray & Castel, 2011).

The relatively poor (high) mBrier scores in OAs remained fairly stable across all rounds. This lack of adjustment corresponds with past studies, which found that OAs may need more time to adjust their strategy on cognitive tasks (Rogers, Hertzog & Fisk, 2000; Touron, Hoyer & Cerella, 2004). However, since the present study only examined one administration of the task that consisted of only five rounds, it may not have been a long enough session for OAs to adjust their strategy.

Based on the mBrier score clusters, OAs were also more likely than the YAs to be in the lower performing clusters (Cluster 2 in the two-cluster analysis and Clusters 2 and 3 in the three-cluster analysis). Across the five rounds, the bets/JOLs of Cluster 2 appeared to decrease from 56.96 in Round 1 to 33.92 in Round 5. This slight decrease in bets/JOLs across rounds for participants in Cluster 2 could be related to the UWP effect previously demonstrated with traditional calibration scores (e.g., Koriat et al., 2002; Stine-Morrow et al., 2006; Tauber & Rhodes, 2012). However, unlike a previous study that showed OAs to be less likely than YAs to experience the UWP effect (Rast & Zimprich, 2009), OAs in our study were *more* likely to be in the Cluster 2 that exhibited the UWP effect.

Based on strategy cluster analysis, two distinct strategy groups emerged. Groups either bet high on average, with high variance (high–high group; meaning that there were bigger discrepancies between their high and low bets), or they bet low on average with low variance (low–low group). The distribution of OAs and YAs between these clusters were not significantly different. This is consistent with the fact that *on average*, there was no difference between OAs and YAs in terms of bets. The lack of age difference among the strategy clusters in our study aligns with previous studies that showed similar metacognitive monitoring abilities between YAs and OAs (see Hertzog & Dunlosky, 2011 for a discussion). For details about the mean and variability of judgement accuracy by age, see Table 4 and Figure 2.

Strategy use did not predict differences in mBrier scores for the two clusters. However, there were significant differences between the three strategy clusters, indicating that a cyclical pattern of bets may be indicative of better mBrier scores. Differences in strategy use were also linked with differences in raw memory ability (i.e. different strategies were associated with differential amounts of correctly recalled words), suggesting that certain strategies might be more preferred by individuals depending on their memory capacities. Knowing which strategies might lead to better recall or MM performance might help inform metacognitive interventions in the future.

It is interesting to note that those who have the highest memory abilities, as measured by recall accuracy, did not necessarily have the best MM abilities, as measured by the mBrier score. Specifically, in the three-strategy cluster analysis, the high-high (Cluster 2) group had the highest aggregate bets and words correct, but they had lower mBrier scores than the cyclical (Cluster 1) group. This contrast also illustrates the value of our mBrier score. If one were to only examine participants’ performances at the word list level, the aggregate information of high bets and high accuracy would suggest a pretty good metamemory. However, the mBrier score was able to tease out the performance at the word level and show a particularly useful strategy. Those who utilized the cyclical strategy of betting high in the beginning of each round (for the first few words that appeared on the list) were indeed better able to remember the words that appeared at the beginning of each list. These participants—despite having lower recall accuracy or memory abilities— were able to adopt a strategy that maximized their metamemory performance. It is yet unclear whether the cyclical strategy is a proactive strategy to lower the amount of effort needed to perform the task of maximizing metamemory performance (i.e., the poorer recall accuracy was an artifact to match their JOL bet ratings) or if it may be a compensatory strategy adopted by participants who realized that their memory might not be as good (i.e., JOL bet ratings were adjusted to match their accurately perceived memory). However, as the Radar graph (Figure 5) shows, the second scenario may be likely as the cyclical pattern became more obvious in later rounds.

These patterns should be examined further in future studies to see if they are consistent across different datasets. Since unsupervised techniques are often “more art than science,” standard validation techniques used in supervised Machine Learning are not able to be applied in the same way. While these clusters match the authors’ anecdotal experience with this task, further confirmation in other datasets is required in order to confirm validity across different samples.

An important contribution of our study is the advancement in MM scoring and the analysis approach. While raw MM score provides insight into both memory capacity and how well MM matches memory performance, it is useful to look at these two components separately. Using a mBrier score as calculated here in addition to a raw MM score allows for a reduced impact of memory capacity on measures of MM. As participants may not view or use the 0 to 10 scale the same way, especially since the instructions to this specific task do not ask the user to treat the 0–10 scale as a probability, ranking the bets before calculating the Brier score lessens the impact of scale usage. Participants who used the scale differently (e.g. someone who is risk averse and only bet between 0 and 5 points, but bet higher on words they remembered compared to someone who bet between 0 and 10 points but also bet higher on words they remembered) were treated the same. Our mBrier score is similar to relative accuracy (resolution) in the MM literature since it is a measure of whether the bet magnitude is different between words that are and are not remembered, regardless of the average magnitude of the bets. However, it allows for the use of the word-by-word numeric JOLs rather than relying on overall correlation, the typical way resolution is measured. It is important to note that our score is not intended to replace other established measures such as Gamma, but to provide a hybrid measure that specifically benefits this task and analysis which require scores to be computable for most or all trials. Looking at individual (non-hybrid measures) is also an important part of assessing metamemory.

While our mBrier score is an improvement on the raw MM score described in the task and shown to the participants, further improvements can still be made. Simulations may be especially useful in the quest to examine the impact of memory capacity on mBrier score by allowing for the exploration of common patterns of betting and word recall, as well as the opportunity to explore hypothetical strategies that maximize mBrier scores depending on memory ability. This rich set of data would benefit from more complex methods that can take into account word and order characteristics such as the length, valance, or position of the word in the list. Most importantly, future work will look into uncovering which strategies are best for whom, exploring individual difference variables such as baseline memory ability, verbal skills, executive functioning, or personality factors, such as need for cognition or self-efficacy, and examine whether the age-related differences in strategy are mainly due to reduced memory capacity, or whether it is primarily driven by these other factors. In the present study, our use of machine-learning approaches introduces a novel and statistically sophisticated method to the study of MM in OA, which we hope will allow for the continued exploration of the mechanisms involved in age-related cognitive changes, with the ultimate goal of developing strategies and interventions to support this population.

## Supplementary Material

Appendix

## Figures and Tables

**Figure 1.**
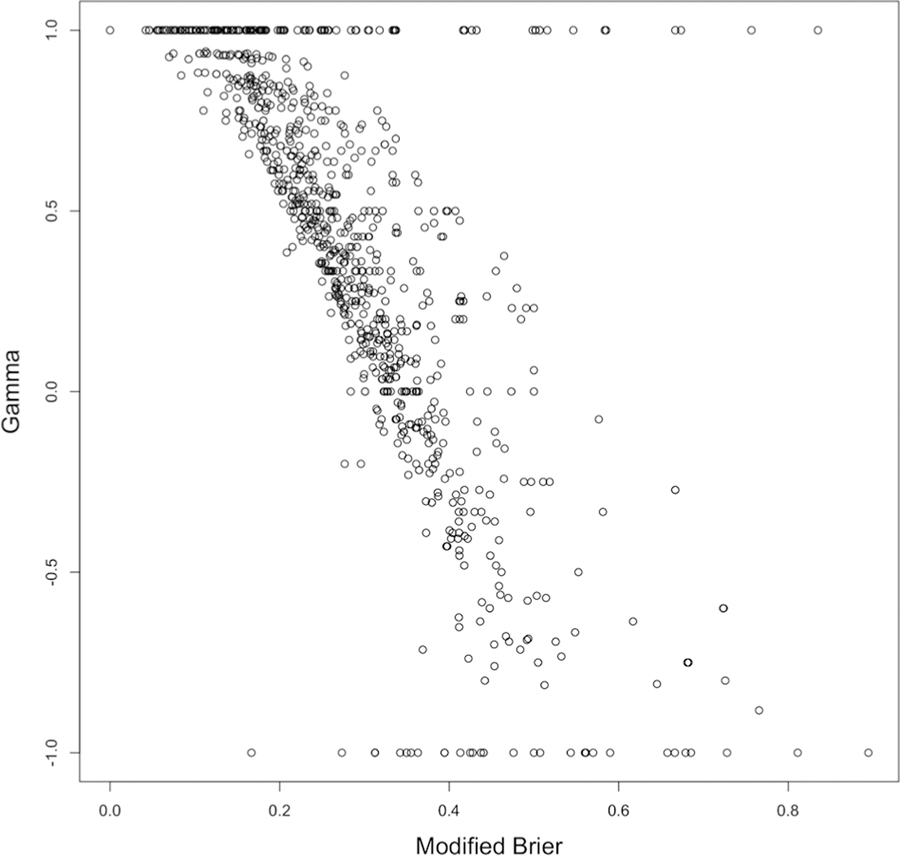
Scatterplot showing relationship between Gamma scores and Modified Brier scores for this sample of data.

**Figure 6.**
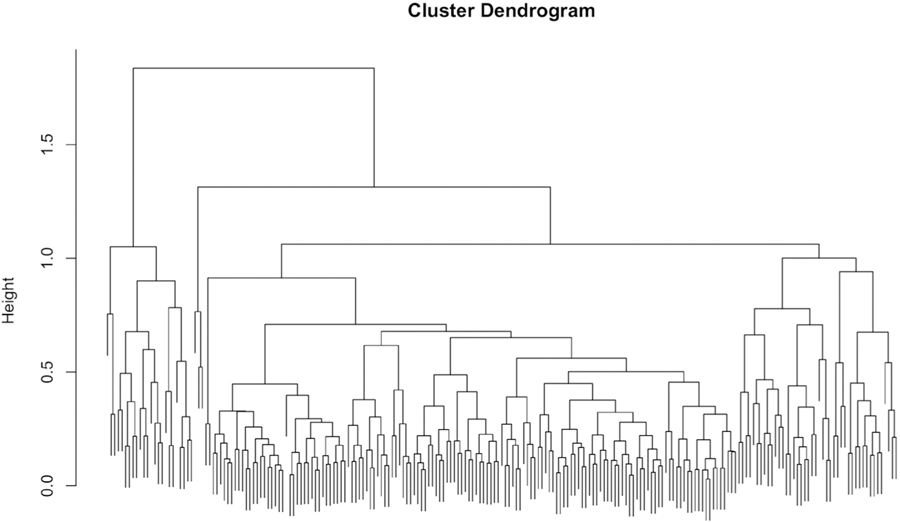
Dendrogram from hierarchical clustering of mBrier score data on the participant level. The distance between clusters (Height) visually represents how different clusters are from one another, whereas the hierarchical arrangement shows how clusters are nested within one another.

**Table 1.** Vignette 1 – Example of when Gamma and mBrier lead to different results.

Participant	word	a	b	c	d	e	f	g	h	i	j	k	l	gamma	mBrier
**A**	**bet (JOL)**	10	10	10	10	10	10	5	10	10	10	10	10	1	0.585
	**accuracy**	1	1	1	1	0	0	0	0	0	0	0	0		
**B**	**bet (JOL)**	10	10	3	3	3	1	1	1	1	2	1	1	1	0.183
	**accuracy**	1	1	1	0	0	0	0	0	0	0	0	0		

**Table 2.** Vignette 2 – Example of when Gamma and mBrier lead to different results.

Participant	word	b	c	d	e	f	g	h	i	j	k	l	gamma	mBrier
**C**	**bet (JOL)**	1	1	4	3	4	4	4	3	4	3	3	−1	0.679
	**accuracy**	1	1	0	0	0	0	0	0	0	0	0		
**D**	**bet (JOL)**	5	4	1	3	1	2	10	6	5	5	10	−1	0.57
	**accuracy**	1	1	1	1	1	1	0	0	0	0	0		

**Table 3.** Vignette 3 – Example of where Brier and mBrier scores differ.

Participant	word	a	b	c	d	e	f	g	h	i	j	k	l	Brier	mBrier
**E**	**bet (JOL)**	4	4	4	4	4	2	2	2	2	2	2	2	0.23	0.18
	**accuracy**	1	1	1	1	0	0	0	0	0	0	0	0		
**F**	**bet (JOL)**	10	10	10	10	10	1	1	1	1	1	1	1	0.09	0.18
	**accuracy**	1	1	1	1	0	0	0	0	0	0	0	0		

**Table 4.** Summary Statistics for OAs and YAs.

	N	Bets (per list)	Words Recalled (per list)	mBrier (per list)
**OAs**	79	4.12 (2.99)	3.67 (1.98)	0.44 (0.23)
**YAs**	138	4.60 (3.39)	5.99 (1.94)	0.29 (0.14)

*Note.*The average scores across all lists (12 words per list) for bets (JOLs), words recalled, and mBrier score are provided.

Standard deviations are given in parentheses.

## References

[R1] Balota DA, Yap MJ, Cortese MJ, Hutchison KA, Kessler B, Loftis B, Neely JH, Nelson DL, Simpson GB, & Treiman R (2007). The English Lexicon Project. Behavior Research Methods, 39(3), 445–459. 17958156 10.3758/bf03193014

[R2] Batchelor JH, & Miao C (2016). Extreme response style: A meta-analysis. Journal of Organizational Psychology, 16(2), 51–62.

[R3] Belmont JM, & Borkowski JG (1988). A group-administered test of children’s metamemory. Bulletin of the Psychonomic Society, 26(3), 206–208.

[R4] Bird H, Franklin S, & Howard D (2001). Age of acquisition and imageability ratings for a large set of words, including verbs and function words. Behavior Research Methods, Instruments & Computers, 33(1), 73–79. doi: 10.3758/BF03195349 11296722

[R5] Bunnell JK, Baken DM, & Richards-Ward LA (1999). The effect of age on metamemory for working memory. New Zealand Journal of Psychology, 28(1), 23–29.

[R6] Connor LT, Dunlosky J, & Hertzog C (1997). Age-related differences in absolute but not relative metamemory accuracy. Psychology and Aging, 12(1), 50. 9100268 10.1037//0882-7974.12.1.50

[R7] Castel AD, Balota DA, & McCabe DP (2009). Memory efficiency and the strategic control of attention at encoding: Impairments of value-directed remembering in Alzheimer’s disease. Neuropsychology, 23(3), 297. 19413444 10.1037/a0014888 PMC2777518

[R8] Castel AD, Benjamin AS, Craik FI, & Watkins MJ (2002). The effects of aging on selectivity and control in short-term recall. Memory & Cognition, 30(7), 1078–1085. 12507372 10.3758/bf03194325

[R9] Chen C, Lee S-Y, & Stevenson HW (1995). Response style and cross-cultural comparisons of rating scales among east Asian and North American students. Psychological Science, 6(3), 170–175. 10.1111/j.1467-9280.1995.tb00327.x

[R10] DeMarie D, & Ferron J (2003). Capacity, strategies, and metamemory: Tests of a three-factor model of memory development. Journal of Experimental Child Psychology, 84(3), 167–193. 12706383 10.1016/s0022-0965(03)00004-3

[R11] Dixon RA, Hultsch DF, & Hertzog C (1988). The metamemory in adulthood (MIA) questionnaire. Psychopharmacology Bulletin, 24(4), 671–688. 3249770

[R12] Dunlosky J, & Connor LT (1997). Age differences in the allocation of study time account for age differences in memory performance. Memory & Cogniti*on*, 25(5), 691–700. 9337587 10.3758/bf03211311

[R13] Dunlosky J, & Hertzog C (1997). Older and younger adults use a functionally identical algorithm to select items for restudy during multitrial learning. The Journals of Gerontology Series B: Psychological Sciences and Social Sciences, 52(4), P178–P186. 10.1093/geronb/52b.4.p178 9224442

[R14] Dunlosky J, & Metcalfe J (2009). Metacognition: A textbook of cognition, educational, life span, and applied psychology Thousand Oaks, CA: Sage.

[R15] Duncan RB (1972). Characteristics of organizational environments and perceived environmental uncertainty. Administrative Science Quarterly, 17(3), 313–327.

[R16] Dunlosky J, & Thiede KW (2013). Metamemory. In Reisberg D (Ed.), The Oxford Handbook of Cognitive Psychology (pp. 283–298). New York: Oxford University Press.

[R17] Emanuel Robinson A, Hertzog C, & Dunlosky J (2006). Aging, encoding fluency, and metacognitive monitoring. Aging, Neuropsychology, and Cognition, 13(3–4), 458–478. 10.1080/13825580600572983 16887783

[R18] Flavell J, Wellman HM, Kail RV, & Hagen JW (1977). Metamemory; Perspective on the development of memory and cognition

[R19] Gilewski MJ, Zelinski EM, & Schaie KW (1990). The Memory Functioning Questionnaire for assessment of memory complaints in adulthood and old age. Psychology and Aging, 5(4), 482. 2278670 10.1037//0882-7974.5.4.482

[R20] Hertzog C, & Dunlosky J (2011). Metacognition in later adulthood: Spared monitoring can benefit older adults’ self-regulation. Current Directions in Psychological Science, 20(3), 167–173. doi: 10.1177/0963721411409026 24478539 PMC3903298

[R21] Hertzog C, Dunlosky J, & Sinclair SM (2010). Episodic feeling-of-knowing resolution derives from the quality of original encoding. Memory & Cognition, 38(6), 771–784. 20852240 10.3758/MC.38.6.771 PMC2943856

[R22] Hertzog C, Kidder DP, Powell-Moman A, & Dunlosky J (2002). Aging and monitoring associative learning: Is monitoring accuracy spared or impaired? Psychology and Aging, 17(2), 209. 12061407

[R23] Kelemen WL, Frost PJ, & Weaver CA III (2000). Individual differences in metacognition: Evidence against a general metacognitive ability. Memory & Cognition, 28(1), 92–107. 10714142 10.3758/bf03211579

[R24] Koriat A (1997). Monitoring one’s own knowledge during study: A cue-utilization approach to judgments of learning. Journal of experimental psychology: General, 126(4), 349.

[R25] Koriat A, & Goldsmith M (1996). Monitoring and control processes in the strategic regulation of memory accuracy. Psychological Review, 103(3), 490–517. 8759045 10.1037/0033-295x.103.3.490

[R26] Koriat A, Ma’ayan H, Sheffer L, & Bjork R (2006). Exploring a mnemonic debiasing account of the underconfidence-with-practice effect. Journal of Experimental Psychology: Learning, Memory, & Cognition, 32(3), 595–608. doi: 10.1037/0278-7393.32.3.595 16719669

[R27] Koriat A, Sheffer L, & Ma’ayan H (2002). Comparing objective and subjective learning curves: judgments of learning exhibit increased underconfidence with practice. Journal of Experimental Psychology: General, 131(2), 147. 12049237

[R28] Lund K, & Burgess C (1996). Producing high-dimensional semantic spaces from lexical co-occurrence. Behavior Research Methods, Instruments, & Computers, 28(2), 203–208.

[R29] Maki RH, Shields M, Wheeler AE, & Zacchilli TL (2005). Individual differences in absolute and relative metacompre-hension accuracy. Journal of Educational Psychology, 97(4), 723–731.

[R30] McGillivray S, & Castel AD (2011). Betting on memory leads to metacognitive improvement by younger and older adults. Psychology and Aging, 26(1), 137. 21417541 10.1037/a0022681

[R31] Metcalfe J (2002). Is study time allocated selectively to a region of proximal learning? Journal of Experimental Psychology: General, 131(3), 349. 12214751 10.1037//0096-3445.131.3.349

[R32] Nelson TO (1984). A comparison of current measures of the accuracy of feeling-of-knowing predictions. Quantitative Methods in Psychology, 95(1), 109–133. 6544431

[R33] Price J, Hertzog C, & Dunlosky J (2010). Self-regulated learning in younger and older adults: Does aging affect metaco-gnitive control? Aging, Neuropsychology, and Cognition, 17(3), 329–359. 10.1080/13825580903287941 PMC3197275 19866382

[R34] Price J, & Murray RG (2012). The region of proximal learning heuristic and adult age differences in self-regulated learning. Psychology and Aging, 27(4), 1120. 22946525 10.1037/a0029860

[R35] Rast P, & Zimprich D (2009). Age differences in the underconfidence-with-practice effect. Experimental Aging Research, 35(4), 400–431. 20183099 10.1080/03610730903175782

[R36] Rhodes MG, & Tauber SK (2011). The influence of delaying judgments of learning on metacognitive accuracy: A meta-analytic review. Psychological Bulletin, 137(1), 131. 21219059 10.1037/a0021705

[R37] Rogers WA, Hertzog C, & Fisk AD (2000). An individual differences analysis of ability and strategy influences: Age-related differences in associative learning. Journal of Experimental Psychology: Learning, Memory, and Cognition, 26(2), 359. 10.1037//0278-7393.26.2.359 10764101

[R38] Rousseeuw PJ (1987). Silhouettes: a graphical aid to the interpretation and validation of cluster analysis. Journal of Computational and Applied Mathematics, 20, 53–65.

[R39] Rufibach K (2010). Use of Brier score to assess binary predictions. Journal of clinical epidemiology, 63(8), 938–939. 20189763 10.1016/j.jclinepi.2009.11.009

[R40] Schraw G (2009). A conceptual analysis of five measures of metacognitive monitoring. Metacognition Learning, 4, 33–45. doi: 10.1007/s11409-008-9031-3

[R41] Schwartz BL, & Efklides A (2012). Metamemory and memory efficiency: Implications for student learning. Journal of Applied Research in Memory and Cognition, 1(3), 145–151.

[R42] Souchay C, & Isingrini M (2004). Age related differences in metacognitive control: Role of executive functioning. Brain and Cognition, 56(1), 89–99. 15380879 10.1016/j.bandc.2004.06.002

[R43] Stine-Morrow EA, Shake MC, Miles JR, & Noh SR (2006). Adult age differences in the effects of goals on self-regulated sentence processing. Psychology and Aging, 21(4), 790. 17201498 10.1037/0882-7974.21.4.790 PMC2248724

[R44] Tauber SK, & Rhodes MG (2012). Multiple bases for young and older adults’ judgments of learning in multitrial learning. Psychology and Aging, 27(2), 474. 21942899 10.1037/a0025246

[R45] Tauber SK, & Witherby AE (2015). Metacognition in older adulthood. Encyclopedia of Geropsychology, 1–15.

[R46] Thompson WB, & Mason SE (1996). Instability of individual differences in the association between confidence judgments and memory performance. Memory & Cognition, 24(2), 226–234. 8881325 10.3758/bf03200883

[R47] Touron DR, Hoyer WJ, & Cerella J (2004). Cognitive skill learning: age-related differences in strategy shifts and speed of component operations. Psychology and Aging, 19(4), 565. 15584783 10.1037/0882-7974.19.4.565

[R48] Troyer AK, & Rich JB (2002). Psychometric properties of a new metamemory questionnaire for older adults. The Journals of Gerontology Series B: Psychological Sciences and Social Sciences, 57(1), P19–P27. 10.1093/geronb/57.1.p19 11773220

[R49] Ward JH Jr (1963). Hierarchical grouping to optimize an objective function. Journal of the American Statistical Association, 58(301), 236–244.

[R50] Warriner AB, Kuperman V, & Brysbaert M (2013). Norms of valence, arousal, and dominance for 13,915 English lemmas. Behavior Research Methods, 45(4), 1191–1207. 23404613 10.3758/s13428-012-0314-x

