# Supplementary material for: Exploring Age-Related Metamemory Differences using Modified Brier Scores and Hierarchical Clustering
Source: Open Psychol. Author manuscript; Available in PMC 2021 Mar 9. (PMC7943181; doi:10.1515/psych-2018-0015)
Supplement: Appendix [file NIHMS1574652-supplement-Appendix.pdf]

Appendix

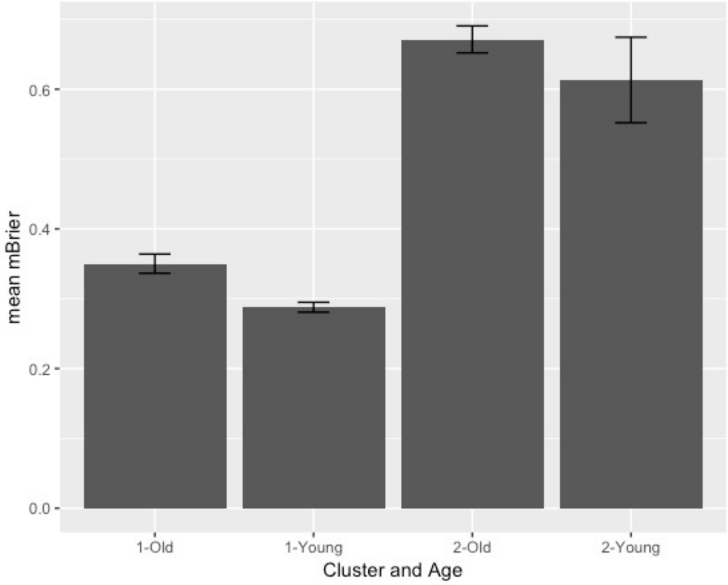

Figure S1. A barplot of the mean mBrier score for each mBrier Cluster (2 clusters), split by age. This plot shows that which OAs and YAs may still score differentially, similar patterns emerge between OAs and YAs with Cluster 1 having lower mBrier scores, and Cluster 2 having higher mBrier scores for both Age groups.

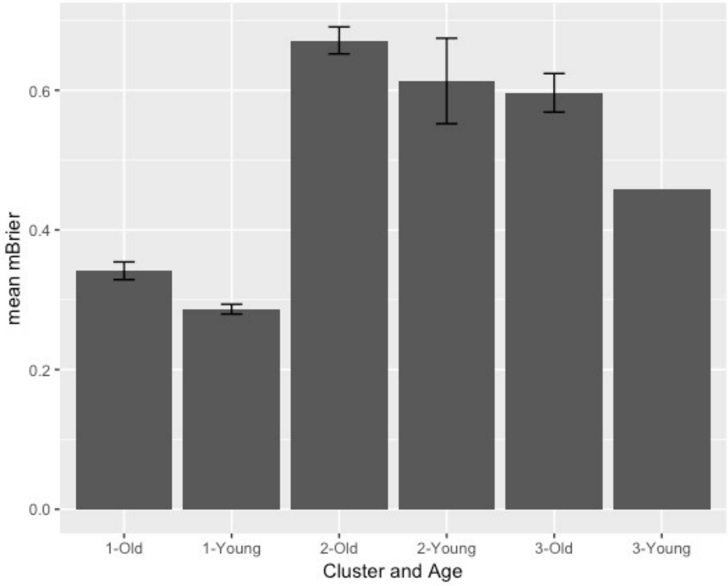

Figure S2. A barplot of the mean mBrier score for each mBrier Cluster (3 clusters), split by age. This plot shows that which OAs and YAs may still score differentially, similar patterns emerge between OAs and YAs with Cluster 1 having lower mBrier scores, Cluster 2 having higher mBrier scores, and Cluster 3 having scores in the middle for both Age groups.

Brought to you by | University of California - Irvine
Authenticated
Download Date | 7/1/19 12:37 AM

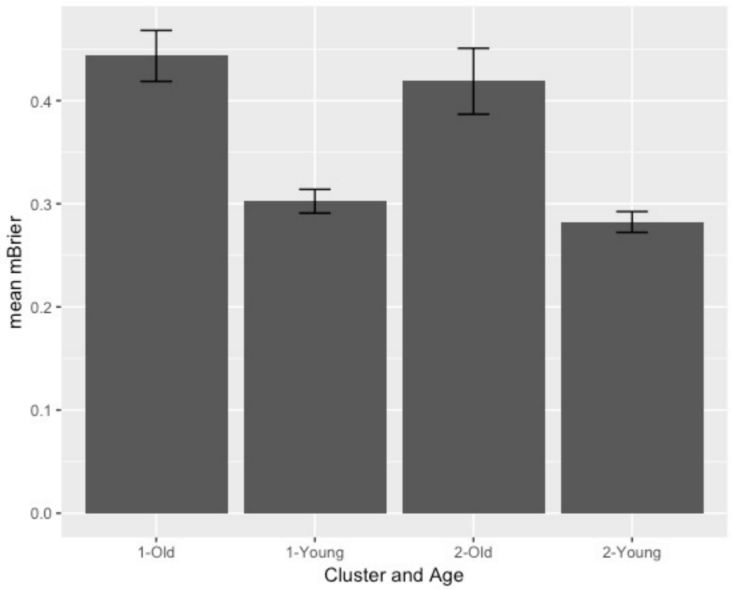

Figure S3. A barplot of the mean mBrier score for each Strategy Cluster (2 clusters), split by age. This plot shows that which OAs and YAs may still score differentially, similar patterns emerge between OAs and YAs with scores being similar (but slightly lower for Cluster 2) for both Age groups.

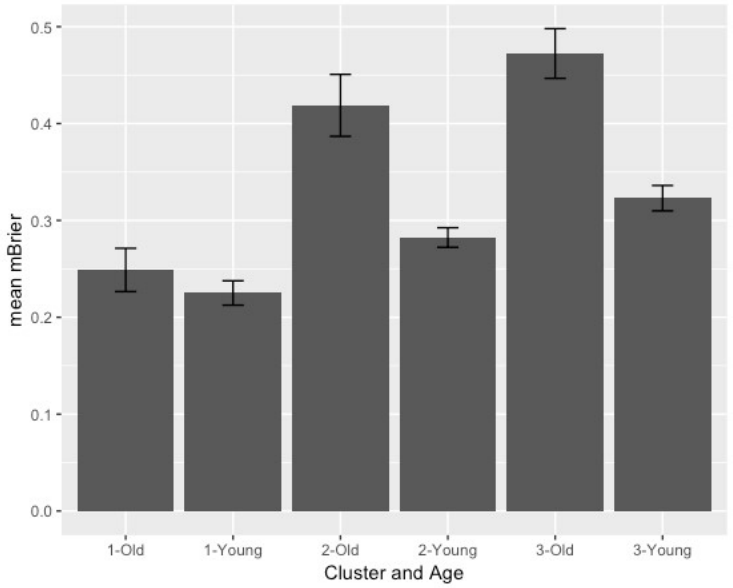

Figure S4. A barplot of the mean mBrier score for each Strategy Cluster (3 clusters), split by age. This plot shows that which OAs and YAs may still score differentially, similar patterns emerge between OAs and YAs with Cluster 1 having lower mBrier scores, Cluster 2 having midrange mBrier scores, and Cluster 3 having higher mBrier scores for both Age groups.

Brought to you by | University of California - Irvine
Authenticated
Download Date | 7/1/19 12:37 AM

Table S1. Summary Statistics for Wordlist Characteristics.

	Concreteness		Imageability		Length	
	A	B	A	B	A	B
Valid	60	60	19	60	60	60
Mean (sd)	4.65(0.52)	3.59 (0.90)	557.3 (69.01)	441.5(66.18)	4.28 (0.69)	5.02 (1.02)
Minimum	1.33	1.7	348	331	3	4
Maximum	5	4.96	643	534	5	7
	**Arousal**		**Valence**		**Log Freq**	
	A	B	A	B	A	B
Valid	60	59	59	60	60	60
Mean (sd)	3.99 (0.92)	3.92 (0.82)	6.00(0.97)	5.52(0.71)	9.42(1.29)	10.31(1.15)
Minimum	2.35	1.67	3.63	4.17	5.27	8.55
Maximum	7.24	5.75	7.89	6.95	12.16	12.72

	Polysemy	
	A	B
Valid	25	20
Mean (sd)	3.48 (3.24)	5.3 (4.37)
Minimum	1	1
Maximum	15	19

Brought to you by | University of California - Irvine
Authenticated
Download Date | 7/1/19 12:37 AM
